# B Cell dysfunction in tumor-draining lymph nodes predicts relapse in oral squamous cell carcinoma

**DOI:** 10.1007/s00262-025-04231-9

**Published:** 2025-11-12

**Authors:** Vilma Liljeström, Pedro Farrajota Neves da Silva, Rusana Bark, Alexandra Elliot, Linda Marklund, Gregori Margolin, Susanna Kumlien Georén, Lars-Olaf Cardell, Krzysztof Piersiala

**Affiliations:** 1 Division of ENT Diseases, Department of Clinical Sciences, Intervention and Technology, Karolinska Institutet, Stockholm, Sweden; 2 Department of Otorhinolaryngology, Karolinska University Hospital, Stockholm, Sweden; 3 Department of Pathology and Cancer Diagnostics, Karolinska University Hospital, Stockholm, Sweden; 4 Medical Unit Head Neck, Lung and Skin Cancer, Karolinska University Hospital, Stockholm, Sweden; 5 Department of Surgical Sciences, Section of Otolaryngology and Head and Neck Surgery, Uppsala University, Uppsala, Sweden

**Keywords:** Oral squamous cell carcinoma, Tumor-draining lymph nodes, B cells, Prognosis, Flow cytometry, Recurrence

## Abstract

Oral squamous cell carcinoma (OSCC) presents a persistent clinical challenge, with high recurrence rates and limited improvements in survival despite therapeutic advances. Tumor-draining lymph nodes (TDLNs) are key immunological sites where anti-tumor responses are orchestrated, yet the prognostic relevance of B cell phenotypes in TDLNs remains underexplored. TDLNs from 49 OSCC patients treated at Karolinska University Hospital were prospectively analyzed. Single-cell suspensions were examined using multicolor flow cytometry to characterize B cell subsets (naïve, memory, plasma cells) and expression of immunoregulatory markers (CD11c, CD24, CXCR5, CD73, HLA-DR, PD-L1). B cell profiles were correlated with clinical outcomes, including disease-free survival (DFS) and overall survival (OS). Disease-free patients exhibited a distinct B cell profile marked by a higher proportion of naïve B cells, strong CXCR5 and CD11c expression, and increased plasma cell differentiation. Recurrence was associated with elevated CD24, CD73, and HLA-DR expression, markers linked to immunoregulatory or dysfunctional B cell states. Interestingly, high PD-L1 expression on memory B cells correlated with improved prognosis, suggesting a context-dependent immune function. In line with these observations, multivariate analysis confirmed HLA-DR expression, together with nodal status, as independent prognostic factors for survival in OSCC. B cell phenotypes in OSCC TDLNs are strongly associated with patient outcomes. A microenvironment enriched in naïve and functionally active B cells supports durable tumor control, whereas regulatory/exhausted phenotypes are linked to recurrence. These findings position B cell markers as promising prognostic indicators and therapeutic targets in OSCC, warranting validation in larger cohorts and functional studies.

**Supplementary Information:**

The online version contains supplementary material available at 10.1007/s00262-025-04231-9.

## Introduction

Oral squamous cell carcinoma (OSCC) is among the most prevalent malignancies of the head and neck (1). Despite advances in surgical techniques, radiotherapy, and systemic therapies, overall survival remains suboptimal, with 5-year survival rates persistently at 50–60% (2). A key driver of this poor prognosis is the high recurrence rate: approximately 40–50% of patients develop locoregional relapse or distant metastasis within five years (2). Particularly concerning is early recurrence after surgery, which is associated with mortality rates approaching 87% (3).

Cervical lymph node metastasis at diagnosis is a strong negative prognostic factor, nearly halving survival compared to node-negative cases (4). Tumor-draining lymph nodes (TDLNs), the primary sites of tumor antigen presentation to the adaptive immune system, are increasingly recognized as critical immunological checkpoints. However, conventional prognostic models based on tumor size, nodal status, and histopathological features often fail to predict individual outcomes accurately, highlighting the need for improved immunological biomarkers.

Immune checkpoint blockade (ICB), particularly targeting the PD-1/PD-L1 axis, has revolutionized treatment for several solid tumors. Yet, only ~ 20% of OSCC patients experience durable benefit from ICB (5, 6), indicating that both intrinsic and extrinsic immunosuppressive mechanisms may impair anti-tumor immunity. Notably, OSCC TDLNs are enriched in regulatory T cells (Tregs) expressing checkpoint molecules such as PD-1 and CTLA-4 (7), further supporting the role of the TDLN immune landscape in modulating disease progression.

Among immune subsets, B cells remain underexplored in cancer immunology, despite representing a substantial proportion of lymph node-resident immune cells. Once considered primarily antibody producers, B cells are now recognized to perform additional roles, such as cytokine secretion and antigen presentation. Critically, certain B cell subsets, such as regulatory B cells (Bregs), can exert immunosuppressive effects. In OSCC, Bregs enriched in TDLNs produce IL-10 and express inhibitory molecules, including PD-L1 and CD24 (8–11). This functional duality, capable of both anti-tumor and pro-tumor activity, positions B cells as key, yet understudied, modulators of tumor immunity.

In this study, we characterized B cell phenotypes in TDLNs from OSCC patients, focusing on their maturation status (naïve vs. memory), plasma cell differentiation, and expression of immunoregulatory markers (CD11c, CD24, CXCR5, CD73, HLA-DR, PD-L1). We hypothesized that distinct B cell signatures in TDLNs may correlate with recurrence risk and serve as prognostic biomarkers in OSCC.

## Method

### Patients and samples

Patients were prospectively enrolled based on the following inclusion criteria: (1) histologically confirmed primary OSCC; (2) treatment at Karolinska University Hospital, Stockholm, Sweden, between March 2019 and June 2022, involving tumor excision combined with either sentinel node biopsy or sentinel node-assisted elective neck dissection; and (3) informed consent to participate in the study.

Sentinel node (TDLN) identification was performed using SPECT-CT imaging, with intraoperative confirmation via gamma probe detection combined with indocyanine green (ICG) injection followed by visualization under near-infrared light. Details of the sentinel node procedure are described in Kågedal et al. (12).

Exclusion criteria included: (1) systemic autoimmune diseases; (2) current or prior diagnosis of other malignancies, including hematolymphoid neoplasms; and (3) acute or chronic conditions potentially affecting lymph node immune function.

### Tissue handling and cell preparation

Freshly excised TDLNs were immediately transported on ice to the Department of Pathology, where a designated pathologist dissected a portion of each node for research. These samples were transferred into pre-chilled MACS Tissue Storage Solution (Miltenyi Biotec, Bergisch Gladbach, Germany #130–100-008).

Tissue dissociation was performed using the Tumor Dissociation Kit (Miltenyi Biotec, Bergisch Gladbach, Germany #130–095-929), combining mechanical and enzymatic methods. Cell suspensions were filtered through a 100 µm cell strainer (BD Biosciences, Franklin Lakes, NJ, USA #352,360), resuspended in Brilliant Stain Buffer (BD Biosciences, Franklin Lakes, NJ, USA #563,794) at a final concentration of 4 × 10⁷ cells/mL, and subsequently cryopreserved at − 180 °C until further analysis, see supplementary methods.

### TDLN selection criteria

Each OSCC patient typically had 1–5 TDLNs identified. For this study, one TDLN per patient was analyzed. In patients with nodal metastasis, the metastatic node was selected. If multiple nodes were metastasis-positive, the node closest to the primary tumor was chosen, following anatomical priority: level I > level II > level II > level III. In metastasis-negative patients, the TDLN closest to the tumor was likewise prioritized using the same anatomical hierarchy.

### Sample preparation

Cryopreserved single-cell suspensions from selected patients were thawed and stained using the LIVE/DEAD™ Fixable Aqua Dead Cell Stain Kit (Thermo Fisher Scientific, Waltham, MA, USA; #L34957) according to the manufacturer’s protocol and supplementary methods. After washing, cells were incubated with Human Fc Block (BD Biosciences, Franklin Lakes, NJ, USA; #564,220), followed by surface staining with the following antibodies in the dark for 25 min at 4 °C: CD19 (BUV395), CD20 (BUV496), HLA-DR (BUV805), CD24 (BV421), CD38 (BV711), CD27 (PE-Cy7), IgD (APC-H7), CD11c (APC), CD73 (BV786), PD-1 (BB700), PD-L1 (PE-CF594), PD-L2 (PE), TIM-3 (BB515), LAG-3 (APC-R700), CXCR5 (BV605). Detailed information on antibody clones and fluorophores is provided in Supplementary Table 1. Cells were then washed and resuspended in PBS containing 1% paraformaldehyde (HistoLab, Gothenburg, Sweden; #02178) for acquisition.

Samples were analyzed on a BD LSRFortessa™ flow cytometer RRID: SCR_018655 (BD Biosciences), and data were processed using FlowJo™ software, version 10.8.1 (BD Life Sciences, Ashland, OR, USA). Gating strategies are shown in Supplementary Figs. 1 and 2.

### Follow-up

All patients underwent structured follow-up. Clinical evaluations were performed every three months during the first two years after treatment (surgery with or without postoperative radiotherapy or chemoradiotherapy), and every six months from years three to five. Median follow-up time was 34.8 months, with 5 to 52 months in range.

Disease-free survival (DFS) was defined as the interval from the end of treatment to the first recurrence, death from any cause, or the last follow-up. Overall survival (OS) was defined as the time from the end of treatment to cancer-related death or the most recent follow-up.

### Statistical analysis

Normality was assessed using the D’Agostino & Pearson test. For normally distributed data, comparisons between groups were performed using a t-test (recurrence status) or two-way ANOVA (B cell phenotypes and recurrence status). For non-normally distributed, unpaired data, the Mann–Whitney U test was used. Kaplan–Meier curves were generated, and survival distributions were compared using the log-rank test. Statistical analyses were performed with GraphPad Prism version 10.8.1 RRID: SCR_002798 (GraphPad Software, La Jolla, CA, USA).

Survival analyses were performed using SPSS RRID: SCR_002865 (version 29.0.1.0; SPSS Inc., Chicago, IL, USA). Univariate associations were first assessed by binary logistic regression, followed by multivariate analysis using Cox proportional hazards models to identify independent prognostic factors. Variables with a p-value below 0.02 in the univariate analyses, along with the two treatment-related variables: postoperative radiotherapy and the type of surgical procedure (SNB, neck dissection, or SNLB), were included in the multivariate models. This more stringent cutoff compared to a p-value of 0.05 was applied because a large number of variables (13 variables for DFS and 9 variables for OS) showed significance at the 0.05 level, and given the limited sample size, including all would have reduced the reliability of the multivariate models. To avoid collinearity in the multivariate analyses, dependent variables were not included simultaneously; for example, HLA-DR⁺ B cells and HLA-DR⁺ memory B cells were both significant in univariate analyses, but only the total HLA-DR⁺ B cell population was entered into the multivariate model, since the memory subset is a component of the total population.

Data are presented as mean ± standard deviation (SD). Statistical significance was defined as *p* < 0.05 (*p < 0.05, **p < 0.01, *** *p* < 0.001). Graphs were generated using GraphPad Prism, version 10.0.1 RRID: SCR_002798 (GraphPad Software, La Jolla, CA, USA).

### Ethical approval

All procedures involving human participants complied with the ethical standards of the institutional and national research committees and the 1964 Declaration of Helsinki and its later amendments or comparable ethical guidelines. Written informed consent was obtained from all participants. The study was approved by the Regional Ethical Review Board (approval numbers: 2015/1650–31/2 and 2019–03518).

## Results

### Patient characteristics

A total of 49 patients who met the inclusion criteria were enrolled in this study. A summary of the demographic patient characteristics is presented in Table 1, and complete clinical data for all subjects can be found in Supplementary Table 2.

**Table 1:** Demographic characteristics and clinicopathological data of enrolled patients

Variable	N (%)
*Age*	
Age < 60	16 (32.7)
Age ≥ 60	33 (67.3)
*Sex*	
Female	19 (38.8)
Male	30 (61.2)
*Smoking history*	
Never smoker	21 (42.9)
Previous/current smoker	28 (57.1)
*pT status*	
pT1	14 (28.6)
pT2	17 (34.7)
pT3	12 (24.5)
pT4	6 (12.2)
*pN status*	
N0	28 (57.1)
N +	21 (42.9)
*Tumor site*	
Mobile tongue	37 (75.7)
Gingiva	7 (14.3)
Floor of the mouth	4 (8.2)
Buccal mucosa	1 (2.0)
*Recurrence status*	
Recurrence-free	32 (65.3)
Recurrence	17 (34.7)

Among them, 30 were males (61.2%), and 19 were females (38.8%). The mean age at the time of OSCC diagnosis was 63.9 years, ranging from 23 to 87 years. The distribution of tumors was as follows: 37 patients (75.5%) had tumors in the mobile tongue, 7 (14.3%) in the gingiva, 4 (8.2%) in the floor of the mouth, and 1 (2.0%) in the buccal mucosa. Regarding smoking history, 28 patients (57.1%) reported being current or former smokers, while 21 patients (42.9%) were classified as never-smokers. Twenty-one patients (42.9%) had positive pathological nodal status (pN), including micro-metastases in two patients. The distribution of pathological T status (pT) was as follows: pT1–14 patients (28.6%), pT2–17 patients (34.7%), pT3–12 patients (24.5%), and pT4–6 patients (12.2%). Seventeen patients (34.7%) developed recurrence during the follow-up time.

### Proportional difference between memory and naïve B cells in TDLNs is associated with non-recurrent disease

**Fig. 1:**
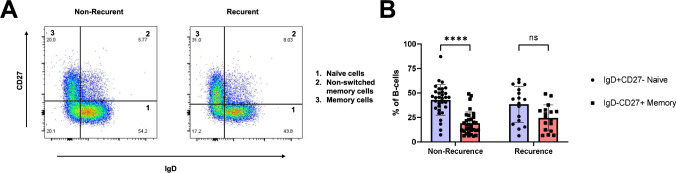
Proportions of naïve and memory B cells in recurrent and non-recurrent patients. Gating strategy for the analysis of naïve and memory B cells based on the expression of CD27 and IgD is presented by two representative dot plots (**A**). Comparison of the proportions between naïve and memory B cells in patients with recurrent (*n* = 17) and non-recurrent disease (*n* = 32) (**B**). A two-way ANOVA with a Sidak multiple comparisons test was performed. Significance is shown by asterisk symbols with significance levels as follows: *p < 0.05, **p < 0.01, ***p < 0.001

The fractions of naïve B cells (CD27⁻IgD⁺) and memory B cells (CD27⁺IgD⁻) were compared between OSCC patients with non-recurrent and recurrent disease. In non-recurrent patients, the proportion of naïve B cells was significantly higher than that of memory B cells (Fig. 1A–B). This difference in B cell differentiation was not observed in patients with recurrent disease (Fig. 1A–B).

### Expression of CD11c, CD24, and HLA-DR on B cells in TDLNs is associated with recurrence status

**Fig. 2:**
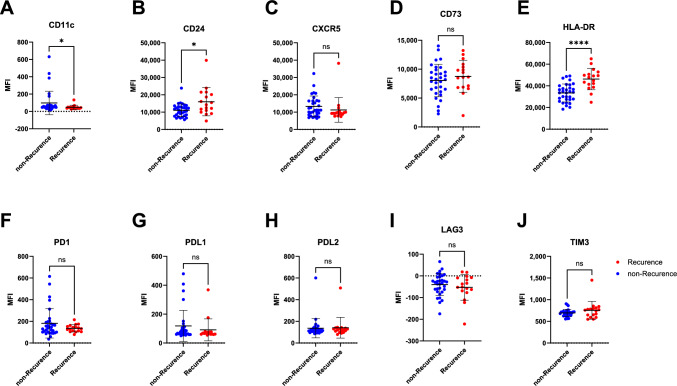
Comparison of surface expression of CD11c (**A**), CD24 (**B**), CXCR5 (**C**), CD73 (**D**), HLA-DR (**E**), PD-1 (**F**), PDL1 (**G**), PDL2 (**H**), LAG3 (**I**) and TIM3 (**J**) on B cells from TDLNs in patients with recurrent (*n* = 17) and non-recurrent disease (*n* = 32). The Mann–Whitney test or t-test was performed depending on normality. Significance is shown by asterisk symbols with significance levels as follows: *p < 0.05, **p < 0.01, ***p < 0.001

Next, we compared the surface expression of CD11c, CD24, CXCR5, CD73, HLA-DR, PD-1, PD-L1, PD-L2, LAG-3, and TIM-3 on B cells from TDLNs in patients with recurrent and non-recurrent disease. Patients with recurrent disease exhibited significantly lower CD11c expression and higher CD24 and HLA-DR expression on total B cells. In contrast, the expression levels of CXCR5, CD73, and the checkpoint molecules (PD-1, PD-L1, PD-L2, LAG-3, and TIM-3) were similar between the two patient groups (Fig. 2). We further analyzed marker expression on naïve and memory B cell subsets. In patients with recurrent disease, memory B cells showed significantly higher CD24 and HLA-DR, and lower PD-1 expression. On naïve B cells, HLA-DR expression was also significantly increased (Supplementary Fig. 3).

### Expression levels of CD11c, CD24 on B cells, and the proportion of Plasma cells in TDLNs, correlate with disease-free survival and overall survival

**Fig. 3:**
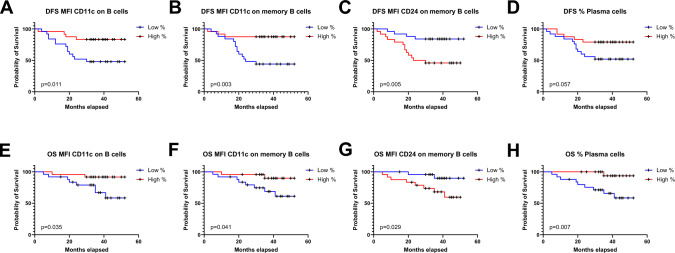
Kaplan–Meier curves for disease-free survival (DFS) (**A**–**D**) and overall survival (OS) (**E**–**H**) in relation to levels of expression of B cells and memory B cells in TDLNs. CD11c expression on B cells (**A**, **E**), CD11c expression on memory B cells (**B**, **F**), CD24 expression on memory B cells (**C**, **G**), and proportion of Plasma cells (**D**, **H**). A log-rank test was performed, and *p* < 0.05 was considered significant

To perform survival analyses, marker expression levels were dichotomized into low and high groups using the median value as the threshold. First, CD11c, a molecule involved in B cell differentiation toward plasma cells, was investigated. In Kaplan–Meier analyses, patients with CD11c expression below the median on both total B cells and memory B cells showed a significantly shorter disease-free survival (DFS) and overall survival (OS) (Fig. 3A, B, E, and F). Conversely, higher expression of CD24 on memory B cells (i.e., above the median) was associated with a significantly worse prognosis, with reduced DFS and OS (Fig. 3C, G). Next, the proportion of plasma cells in TDLNs was assessed. Patients with a lower proportion of plasma cells (below the median) exhibited a significantly shorter OS compared to those with a higher proportion (Fig. 3D, H). A summary of p-values, 3-year DFS, and OS estimates is provided in Supplementary Table 3.

### Expression levels of CXCR5 on B cells and CD73 on naïve B cells in TDLNs correlate with disease-free survival and overall survival

**Fig. 4:**
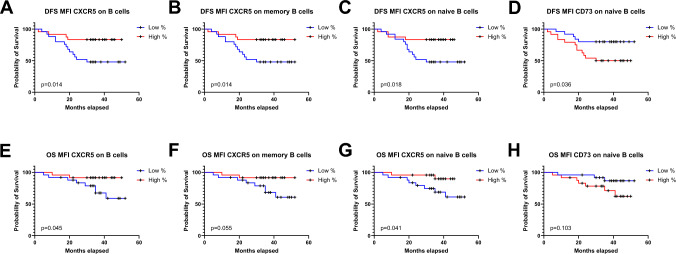
Kaplan–Meier curves for disease-free survival (DFS) (**A**–**D**) and overall survival **(OS)** (**E**–**H**) in relation to levels of expression of B cells, memory B cells, and naïve B cells in TDLNs. CXCR5 expression on B cells **A**, **E**, CXCR5 expression on memory B cells (**B**, **F**), CXCR5 expression on naïve B cells (**C**, **G**), and CD73 expression on naïve B cells (**D**, **H**). A log-rank test was performed, and *p* < 0.05 was considered significant

Next, markers involved in an effective adaptive anti-tumor immune response were analyzed. Low CXCR5 expression on total B cells, memory B cells, and naïve B cells (i.e., below the median) was associated with significantly shorter disease-free survival (DFS) and overall survival (OS), with the exception of OS for memory B cells (Fig. 4A–C, E–G).

Conversely, high expression of CD73, a known pro-tumor marker on naïve B cells, was associated with significantly shorter DFS compared to patients with low expression (Fig. 4D, H).

### Expression levels of HLA-DR on B cells and PDL1 on memory B cells in TDLNs correlate with disease-free survival and overall survival

**Fig. 5:**
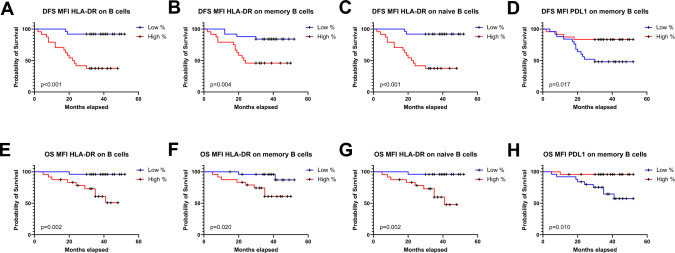
Kaplan–Meier curves for disease-free survival (DFS) (**A**–**D**) and overall survival **(OS)** (**E**–**H**) in relation to levels of expression of B cells, memory B cells, and naïve B cells in TDLNs. HLA-DR expression on B cells (**A**, **E**), HLA-DR expression on memory B cells (**B**, **F**), HLA-DR expression on naïve B cells (**C**, **G**), and PDL1 expression on memory B cells (**D**, **H**). A log-rank test was performed, and *p* < 0.05 was considered significant

Markers involved in the activation and inhibition of B cells, including memory and naïve subsets, were analyzed. Higher expression of the activation marker HLA-DR on total B cells, memory B cells, and naïve B cells was associated with significantly worse outcomes, reflected by shorter disease-free survival (DFS) and overall survival (OS) (Fig. 5A, B, C, E, F, and G). Lower expression of PD-L1 on memory B cells was also associated with significantly shorter DFS and OS (Fig. 5D and H).

### HLA-DR expression and nodal status are independent prognostic factors for survival in OSCC

Lastly, univariate binary logistic regression analyses were performed for each variable. For relapse, significant associations were observed for a higher T stage (T3–T4 vs. T1–T2, *p* = 0.002), smoking (previous/current vs. never, *p* = 0.034), high expression of HLA-DR⁺ on B cells (*p* < 0.001) low expression of CD11c⁺ on B cells (*p* = 0.013), low expression of CXCR5⁺ on B cells (*p* = 0.013), low expression of CD11c⁺ on memory B cells (*p* = 0.003), high expression of HLA-DR⁺ on memory B cells (*p* = 0.007), high expression of CD24⁺ on memory B cells (*p* = 0.007), low expression of CXCR5^+^ on memory B cells (*p* = 0.013), low expression of PDL1⁺ on memory B cells (*p* = 0.013), high expression of HLA-DR⁺ on naïve B cells (*p* < 0.001), low expression of CXCR5⁺ on naïve B cells (*p* = 0.013), and high expression of CD73⁺ on naïve B cells (*p* = 0.032) which all correlated with recurrence, see supplementary Table 4.

For disease-specific death, significant associations were found for higher T stage (*p* = 0.003), N stage (N + vs. N0, *p* = 0.016), smoking (previous/current vs. never, *p* = 0.033), higher expression of HLA-DR⁺ on B cells (*p* = 0.016), lower proportion of plasma B cells (*p* = 0.020),, higher expression of HLA-DR⁺ on memory B cells (*p* = 0.041), higher expression of CD24^+^ on memory B cells (*p* = 0.041), lower expression of PDL1⁺ on memory B cells (*p* = 0.020) and higher expression of HLA-DR^+^ on naive B cells (*p* = 0.016) which all associated with disease-specific death, see supplementary Table 4.

Variables with *p* < 0.02 were subsequently entered into multivariate Cox proportional hazards models for DFS and OS. In the multivariate analyses, high HLA-DR expression remained an independent prognostic factor for both DFS (HR 7.05, 95% CI 1.30–38.15, *p* = 0.023) and OS (HR 13.32, 95% CI 1.34–132.50, *p* = 0.027). In addition, nodal status (N + vs. N0) was independently associated with OS (HR 8.25, 95% CI 1.21–56.40, *p* = 0.031) (Table 2).

**Table 2:** Multivariate regression analyses for disease-free survival (DFS) and overall survival (OS). Results from Cox proportional hazards models are shown, including parameters, variables, significance levels (Sig.), hazard ratios [Exp(B)], and corresponding 95% confidence intervals (CI). The variable stated first is the reference variable. Significant values are marked in bold

	Parameter	Variable	Sig	Exp(B)	95% CI lower	95% CI upper
DFS	T-stage	T1 + T2 vs T3 + 4	0.654	1.321	0.391	4.464
	HLA-DR^+^ B cells	Low vs high	**0.044**	5.728	1.049	31.287
	CD24^+^ memory B cells	Low vs high	0.16	2.443	0.702	8.499
	PDL1^+^ memory B cells	Low vs high	0.859	1.099	0.389	3.108
	CD11c^+^ B cells	Low vs high	0.481	0.628	0.172	2.292
	CXCR5^+^ B cells	Low vs high	0.749	0.804	0.211	3.057
	Postoperative radiotherapy	No vs yes	0.315	2.034	0.509	8.135
	SNB or neck + SNLB	SNB vs neck + SNLB	0.261	0.556	0.2	1.546
OS	T-stage	T1 vs T34	0.637	1.59	0.232	10.883
	HLA-DR^+^ B cells	Low vs high	**0.027**	13.322	1.339	132.501
	PDL1^+^ memory B cells	Low vs high	0.59	0.502	0.041	6.169
	Postoperative radiotherapy	No vs yes	0.968	0.003	0	1.1E + 104
	SNB or Neck + SNLB	SNB vs neck + SNLB	0.296	1.481	0.709	3.095
	N-stage	N0 vs N +	**0.031**	8.249	1.206	56.403
	Proportion of plasma B cells	Low vs high	0.081	0.093	0.006	1.338

## Discussion

This study identifies distinct phenotypic features of B cells within TDLNs in OSCC and establishes their associations with clinical outcomes. Patients who remained disease-free exhibited an immunologically "active" TDLN profile. This profile was characterized by a predominance of naïve B cells, strong CXCR5 and CD11c expression, and higher proportions of plasma cells. In contrast, patients who developed recurrence showed enrichment in markers linked to immunosuppression or dysfunction, including CD24, CD73, and HLA-DR. Notably, higher PD-L1 expression on memory B cells correlated with improved prognosis, suggesting a context-dependent, possibly inflammation-induced, expression profile. These observations collectively suggest that the functional state and composition of B cells in the lymphatic tumor microenvironment contribute to long-term disease control. Furthermore, multivariate analysis confirmed HLA-DR expression on B cells in TDLNs as an independent prognostic factor for DFS and OS in OSCC.

Our analysis of naïve-to-memory B cell ratios highlights a key difference between TDLNs of patients with and without recurrence. In non-recurrent patients, a higher proportion of naïve B cells relative to memory B cells was observed. This distribution may reflect a repertoire less impacted by chronic antigen exposure and capable of responding to emerging tumor antigens. In contrast, patients who relapsed showed a more balanced or memory-skewed distribution at the time of surgery. This pattern suggests prior antigen engagement without effective immune resolution. This interpretation aligns with prior work by Piersiala et al. (8), who reported that B cells in OSCC TDLNs are more naïve and immunosuppressive than in non-tumor-draining nodes and are enriched in IL-10-producing regulatory B cells. Similar findings were reported by Shalapour et al. (13) in hepatocellular carcinoma, where regulatory B cells promoted tumor progression via IL-10-mediated suppression of T cell function. In contrast, Affara et al. described B cells enhancing immunity through tertiary lymphoid structure formation in breast cancer (14), underscoring the context-dependent roles of B cells in cancer.

CXCR5 and CD11c emerged as two markers strongly associated with improved disease-free and overall survival. CXCR5 guides B cells and T follicular helper cells into lymphoid follicles and is essential for germinal center (GC) formation. High CXCR5 expression on both naïve and memory B cells likely indicates active follicular interactions and robust GC activity. In the tumor context, this may translate into improved antibody affinity maturation, enhanced plasma cell generation, and more effective antigen presentation. Piersiala et al. (7) have shown that TDLNs enriched in CXCR5+CD4+T cells correlate with favorable survival, highlighting the importance of intact T–B cell collaboration within GCs. Similarly, Gu-Trantien et al. (15) demonstrated that CXCR5+B cells in tertiary lymphoid structures are associated with prolonged survival in breast cancer.

CD11c expression on memory B cells was also positively associated with survival. CD11c+B cells often represent a T-bet+subset induced under inflammatory conditions, such as chronic infection or IFN-*γ* stimulation (16, 17). These cells are considered potent antigen-presenting cells and may support extrafollicular immune responses. Their abundance in non-recurrent patients could reflect an activated, Th1-biased B cell state primed for plasma cell differentiation or T cell crosstalk (18). Engelbrecht et al. reported CD11c+B cells in melanoma sentinel lymph nodes, suggesting a role in anti-tumor immunity (19). Conversely, in chronic lymphocytic leukemia, CD11c expression has been linked to atypical immunophenotypes and may indicate a state of anergy or exhaustion (20).

High expression of CD24, CD73, and HLA-DR on B cells was associated with shorter survival and earlier recurrence. CD24, typically expressed by immature and transitional B cells, marks regulatory B cell phenotypes capable of IL-10 production and suppression of cytotoxic responses (8). CD73, an ecto-enzyme generating immunosuppressive adenosine, may promote local immune suppression and tumor escape (21). While HLA-DR usually indicates antigen-presenting capacity, its association with poor outcomes in our study may reflect chronic B cell activation or functional exhaustion, impairing effective T cell priming.

High PD-L1 expression on memory B cells emerged as an interesting marker of improved prognosis in our cohort. This finding initially seems counterintuitive, since PD-L1 is classically associated with immune suppression on tumors and antigen-presenting cells. However, PD-L1 upregulation often occurs in the context of interferon-gamma (IFN-*γ*)-rich inflammation and can simply reflect a vigorous immune response (22). Memory B cells in an inflamed tumor microenvironment may actively participate in anti-tumor immunity and can function as potent antigen-presenting cells that restimulate T cells and secrete pro-inflammatory cytokines (e.g., TNF*α*, IL-6, IFN-*γ*) to recruit and activate other immune effectors (23). PD-L1 expression on these B cells may therefore represent an adaptive regulatory mechanism, a “brake” induced by IFN-*γ* during an ongoing immune response, rather than a purely immunosuppressive signal. This contrasts our findings regarding PD-L1 on dendritic cells (DCs) where Hjalmarsson et al. observed that OSCC patients with PD-L1^high^ conventional DCs in tumor‐draining lymph nodes had higher T cell exhaustion and worse survival (24). In that context, PD-L1 on DCs likely inhibits naive T cell priming via PD-1, blunting the development of anti-tumor T cells. In the B cell context, however, PD-L1^+^ memory B cells could denote a feedback checkpoint within an active B/T-cell interaction, without negating the B cells’ pro-immunogenic roles. Supporting this idea, Good-Jacobson et al. found that germinal center B cells upregulate PD-L1 during T-dependent responses (25). Blocking PD-1/PD-L1 signaling in mice led to fewer long-lived plasma cells, indicating that the PD-1 axis actually supports B cell survival and differentiation in germinal centers. Thus, the impact of PD-L1 expression could be highly context- and cell-type-specific: on DCs it may signal a suppressive phenotype that hinders priming, whereas on memory B cells it may mark an IFN-*γ*-stimulated, immunologically active state that aligns with ongoing anti-tumor immunity.

Our contrasting findings seem to be confirmed in a recent meta-analysis. The authors found that high PD-L1 on immune cells corresponded to significantly improved survival in curative-intent HNSCC patients (26). However, the overall heterogeneity of the analysis was substantial (τ^2^ = 0.25, I^2^ = 78.74%), suggesting that the magnitude of the association varied considerably across studies, warranting cautious interpretation and further exploration of underlying sources of variability.

In the univariate analyses, several clinicopathological and B cell-related variables were associated with recurrence and disease-specific death, but in multivariable models, only HLA-DR expression on B cells and nodal status retained independent prognostic value. The adverse association of higher HLA-DR expression on B cells may indicate a state of chronic antigen presentation and immune activation that skews toward dysfunction (similarly to T cell exhaustion) within TDLNs and the TME rather than effective priming. This context-dependent role is consistent with reports that B cells in HNSCC can exert bidirectional, sometimes immunosuppressive effects, and with observations that elevated HLA-DR on activated T cell phenotypes tracks with poorer prognosis in HNSCC, suggesting that heightened MHC class II-mediated activation can coincide with maladaptive immune states(27, 28).

Moreover, potential confounding factors such as treatment modality were also considered. In our cohort, variables including postoperative radiotherapy and the type of surgical approach (sentinel node biopsy versus sentinel node-guided neck dissection) were entered into the univariate and multivariate analyses. Their inclusion did not alter the associations observed for B cell-related variables or clinicopathological parameters in the final models. These findings suggest that treatment-related factors did not substantially confound the prognostic impact of immune phenotypes in TDLNs in this study.

Our results build on prior studies of the TDLN immune landscape. Piersiala et al. previously showed that regulatory T cells and Bregs accumulate in OSCC TDLNs and are associated with nodal metastasis and disease progression (7, 8, 29). Our study extends this work by characterizing the B cell compartment in more detail. These findings reinforce the concept that TDLNs function as immune regulatory hubs that shape the anti-tumor response.

The potential of B cell phenotypes in TDLNs to serve as prognostic biomarkers represents a key clinical implication. Assessing expression levels of CD24, CD73, CD11c, or CXCR5 through surgical pathology or flow cytometry could help identify patients at increased risk of recurrence, even in early-stage disease. Such information may inform decisions regarding adjuvant therapy, surveillance strategies, or inclusion in immunotherapy trials. Furthermore, our findings suggest that modulating B cell function either by inhibiting Bregs (e.g., via anti-CD73 agents) or promoting follicular B cell activity could improve clinical outcomes in OSCC. Anti-CD73 agents such as oleclumab are under clinical evaluation. Early-phase trials of oleclumab in combination with PD-1/PD-L1 checkpoint inhibitors have shown encouraging activity in solid tumors, improving anti-tumor immune responses without significant added toxicity (30).

However, targeting B cells broadly requires caution. Depleting Bregs without preserving protective subsets such as CD11c+or CXCR5+memory B cells could impair beneficial immune responses. A more nuanced understanding of the spatial and functional heterogeneity of B cells in TDLNs is therefore essential.

This study has a few limitations that should be acknowledged. First, it was conducted at a single center with a relatively small sample size, which may limit the statistical power and generalizability of the findings. Second, potential confounding factors, such as treatment variations among patients, cannot be fully excluded. Third, the absence of functional assays restricts the ability to directly assess the mechanistic roles of the identified immune cell populations. Taken together, these limitations highlight the need for validation in larger, multi-center cohorts to confirm the robustness and applicability of our conclusions across diverse patient populations. Moreover, one could argue that the observed effects are biased by the TDLN with metastasis and that it is the metastasis alone that creates the observed immunological state. However, when comparing the TDLNs with and without metastasis in patients with relapse showed no significant difference in expression of the surface markers analyzed on B cells, naive B cells, and memory B cells was found. Similarly, the proportion of memory, naïve, and plasma B cells did not differ significantly, see supplementary Fig. 4. In conclusion, this study highlights the prognostic significance of B cell phenotypes in OSCC TDLNs and supports their potential as biomarkers and therapeutic targets. Validation in larger cohorts, mechanistic and functional studies are needed to clarify the role of B cells in regulating anti-tumor immunity.

## Conclusions

In summary, our study demonstrates that B cell phenotypes in OSCC tumor-draining lymph nodes are informative indicators of prognosis. A TDLN immune profile characterized by a predominance of naïve B cells, active plasma cell generation, CXCR5-mediated follicular organization, and CD11c⁺ memory B cell subsets was associated with long-term disease control. In contrast, enrichment of CD24, CD73, and HLA-DR expression marked a potentially dysfunctional or immunosuppressive niche linked to disease recurrence. Importantly, in multivariate models, only HLA-DR expression and nodal status retained independent prognostic significance, highlighting their robustness beyond other clinicopathological and immunological factors. These findings underscore the critical role of TDLNs in anti-tumor immunity and support the utility of B cell-focused profiling as a source of prognostic biomarkers and therapeutic targets. Future research should explore strategies to preserve beneficial B cell functions while limiting immunoregulatory mechanisms within TDLNs to improve OSCC treatment outcomes.

## Supplementary Information

Below is the link to the electronic supplementary material.

Supplementary file1 (DOCX 2148 KB)

## Data Availability

The authors confirm that the data supporting the findings of this study are available within the article. Further information is available from the corresponding author upon request.

## Abbreviations

Bregs: Regulatory B cells
DFS: Disease-free survival
GC: Germinal center
ICB: Immune checkpoint blockade
ICG: Indocyanine green
OS: Overall survival
OSCC: Oral squamous cell carcinoma
pN: Pathological nodal status
pT: Pathological T status
SD: Standard deviation
TDLNs: Tumor-draining lymph nodes
Tregs: Regulatory T cells
