# Supplementary material for: B Cell dysfunction in tumor-draining lymph nodes predicts relapse in oral squamous cell carcinoma
Source: Cancer Immunol Immunother. 2025 Nov 12;74(12):373. doi: 10.1007/s00262-025-04231-9 (PMC12612397; doi:10.1007/s00262-025-04231-9)
Supplement: Supplementary file 1 — Supplementary file1 (DOCX 2148 KB) [file 262_2025_4231_MOESM1_ESM.docx]

**Supplementary Tables, Figures, and Methods**

**Supplementary Table 1.** Antibody panel used for flow cytometry analysis. Panel targeting B cell maturation, checkpoint expression, and activation on B cells.

| **Antigen** | **Fluorochrome** | **Clone** | **Antibody supplier** | **RRID** |
| --- | --- | --- | --- | --- |
| LIVE/DEAD Fixable 780 | APC-Cy7 AmCyan |  | BD | AB_2869673 |
| CD19 | BUV395 | SJ25C1 | BD | AB_2738272 |
| CD20 | BUV496 | 2H7 | BD | AB_3685218 |
| HLA-DR | BUV805 | G46-6 | BD | AB_2872757 |
| CD24 | BV421 | ML5 | BD | AB_2737796 |
| PD-1 CD279 | BB700 | EH12.1 | BD | AB_2744348 |
| CXCR5 | BV605 | RF8B2 | BD | AB_2740110 |
| CD38 | BV711 | HIT2 | BD | AB_2738516 |
| CD73 | BV786 | AD2 | BD | AB_2740928 |
| TIM3 | BB515 | 7D3 | BD | AB_2744368 |
| PD-L2 CD273 | PE | MIH18 | BD | AB_647197 |
| PD-L1 CD274 | PE-CF594 | MIH1 | BD | AB_2738400 |
| LAG-3 CD223 | APC-R700 | T47-530 | BD | AB_2744329 |
| CD27 | PE-Cy7 | M-T271 | BD | AB_1727456 |
| CD11c | APC | B-ly6 | BD | AB_398680 |
| IgD | APC-H7 | IA6-2 | BD | AB_10645792 |

**Supplementary Table 2.** Clinical data for all subjects. Patients who were relapse-free/alive at the last follow-up are denoted with an X. The last follow-up date was December 2023.

| **ID** | **Localization** | **Smoking** | **pT** | **pN** | **Region of the neck** | **Metastatic TDLN (Yes/No)** | **Time to Relapse (months)** | **Time to Death (months)** | **Postoperative radiotherapy** | **SNB or Neck+SNLB** |
| --- | --- | --- | --- | --- | --- | --- | --- | --- | --- | --- |
| 1 | T | Yes | T3 | N2b | 2 | Yes | 19 | 20 | Yes | Neck + SNLB |
| 2 | T | No | T2 | N2c | 3 | No | 18 | X | Yes | Neck + SNLB |
| 3 | T | Yes | T2 | N0 | 2 | No | 24 | X | Yes | Neck + SNLB |
| 4 | T | Yes | T3 | N0 | 1 | No | 30 | 41 | Yes | Neck + SNLB |
| 5 | T | Yes | T3 | N2b | 2 | Yes | 7 | X | Yes | Neck + SNLB |
| 6 | F | Yes | T4a | N3b | 3 | Yes | 20 | 35 | Yes | Neck + SNLB |
| 7 | T | Yes | T3 | N2c | 3 | Yes | 22 | 24 | Yes | SNB |
| 8 | T | Yes | T3 | N0 | 2 | No | 17 | 29 | Yes | Neck + SNLB |
| 9 | T | Yes | T3 | N3b | 2 | Yes | 19 | 19 | Yes | Neck + SNLB |
| 10 | G | Yes | T4a | N0 | 3 | No | 8 | X | Yes | Neck + SNLB |
| 11 | G | Yes | T4a | N2b | 2 | Yes | 2 | 10 | Yes | Neck + SNLB |
| 12 | T | Yes | T2 | N1 | 3 | Yes | 4 | 5 | Yes | Neck + S NLB |
| 13 | F | Yes | T1 | N0 | 1 | No | 12 | X | No | SNB |
| 14 | T | No | T1 | N1 | 1 | Yes | 28 | 35 | Yes | SNB |
| 15 | G | Yes | T4a | N0 | 2 | No | 12 | X | No | SNB |
| 16 | T | No | T1 | N0 | 2 | No | 18 | X | No | SNB |
| 17 | G | Yes | T4a | N0 | 2 | No | 23 | X | Yes | SNB |
| 18 | T | Yes | T1 | N0 | 2 | No | X | X | Yes | Neck + SNLB |
| 19 | G | No | T2 | N0 | 1 | No | X | X | No | SNB |
| 20 | T | No | T1 | N0 | 2 | No | X | X | No | Neck + SNLB |
| 21 | T | No | T1 | N0 | 2 | No | X | X | No | Neck + SNLB |
| 22 | G | No | T1 | N0 | 1 | No | X | X | No | Neck + SNLB |
| 23 | F | Yes | T1 | N0 | 2 | No | X | X | No | SNB |
| 24 | T | No | T2 | N0 | 2 | No | X | X | Yes | Neck + SNLB |
| 25 | T | No | T3 | N0 | 2 | No | X | X | Yes | Neck + SNLB |
| 26 | T | Yes | T2 | N0 | 1 | No | X | X | Yes | Neck + SNLB |
| 27 | T | No | T1 | N0 | 2 | No | X | X | No | SNB |
| 28 | T | Yes | T2 | N0 | 1 | No | X | X | No | Neck + SNLB |
| 29 | T | Yes | T1 | N0 | 2 | No | X | X | No | Neck + SNLB |
| 30 | T | No | T2 | N0 | 2 | No | X | X | No | SNB |
| 31 | T | Yes | T2 | N0 | 3 | No | X | X | Yes | Neck + SNLB |
| 32 | T | Yes | T1 | N0 | 2 | No | X | X | No | SNB |
| 33 | T | No | T3 | N0 | 2 | No | X | X | Yes | Neck + SNLB |
| 34 | T | No | T2 | N0 | 2 | No | X | X | Yes | SNB |
| 35 | T | Yes | T2 | N0 | 2 | No | X | X | Yes | SNB |
| 36 | B | Yes | T2 | N0 | 1 | No | X | X | Yes | Neck + SNLB |
| 37 | T | No | T1 | N0 | 2 | No | X | X | No | Neck + SNLB |
| 38 | T | No | T1 | N2b | 1 | Yes | X | X | Yes | Neck + SNLB |
| 39 | G | Yes | T4a | N1 | 3 | Yes | X | X | Yes | Neck + SNLB |
| 40 | T | No | T1 | N1 | 2 | Yes | X | X | No | Neck + SNLB |
| 41 | F | No | T2 | N0(i+) | 1 | Yes | X | X | Yes | Neck + SNLB |
| 42 | T | No | T2 | N1 | 5 | Yes | X | X | Yes | Neck + SNLB |
| 43 | T | No | T2 | N1 | 2 | Yes | X | X | Yes | Neck + SNLB |
| 44 | T | No | T3 | N2b | 1 | Yes | X | X | Yes | Neck + SNLB |
| 45 | T | Yes | T2 | N0(i+) | 3 | Yes | X | X | Yes | Neck + SNLB |
| 46 | T | Yes | T3 | N1 | 2 | Yes | X | X | Yes | Neck + SNLB |
| 47 | T | Yes | T2 | N2b | 2 | Yes | X | X | Yes | Neck + SNLB |
| 48 | T | Yes | T3 | N1 | 2 | No | X | X | Yes | Neck + SNLB |
| 49 | T | No | T3 | N3b | 2 | Yes | X | X | Yes | Neck + SNLB |

Abbreviations: M – male; F- female; T – mobile tongue, G – gingiva, F – floor of the mouth, B – buccal mucosa

**Supplementary Table 3.** Kaplan Meier analysis for 3 years DFS and OS in relation to expression of TIM3, CD11c, LAG3, HLA-DR, CD24, CXCR5, CD73, PDL1, PDL2, and PD-1 on B cells, memory B cells, and naive B cells in TDLNs. Significant p-values (p<0.05) are highlighted with bold numbers.

| **Variable** | **Level** | **DFS** | | **OS** | |
| --- | --- | --- | --- | --- | --- |
|  |  | **3 years DFS** | **p-value**  **(log-rank test)** | **3 years OS** | **p-value**  **(log-rank test)** |
| Proportion of Memory B cells | Low | 75.0% | 0.246 | 85.0% | 0.427 |
|  | High | 58.6% |  | 75.9% |  |
| Proportion of naive B cells | Low | 66.7% | 0.748 | 80.6% | 0.819 |
|  | High | 61.5% |  | 76.9% |  |
| Proportion of plasma B cells | Low | 52.0% | 0.057 | 64.0% | **0.007** |
|  | High | 79.2% |  | 95.8% |  |
| TIM3^+^ B cells | Low | 72.0% | 0.278 | 80.0% | 0.828 |
|  | High | 58.3% |  | 79.2% |  |
| CD11c^+^ B cells | Low | 48.0% | **0.011** | 68.0% | **0.035** |
|  | High | 83.3% |  | 91.7% |  |
| LAG3^+^ B cells | Low | 60.0% | 0.405 | 80.0% | 0.925 |
|  | High | 70.8% |  | 79.2% |  |
| HLA-DR^+^ B cells | Low | 92.0% | **<0.001** | 96.0% | **0.002** |
|  | High | 37.5% |  | 62.5% |  |
| CD24^+^ B cells | Low | 76.0% | 0.105 | 84.0% | 0.384 |
|  | High | 54.2% |  | 75.0% |  |
| CXCR5^+^ B cells | Low | 48.0% | **0.014** | 68.0% | **0.045** |
|  | High | 83.3% |  | 91.7% |  |
| CD73^+^ B cells | Low | 72.0% | 0.341 | 84.0% | 0.439 |
|  | High | 58.3% |  | 75.0% |  |
| PDL2^+^ B cells | Low | 72.0% | 0.278 | 80.0% | 0.978 |
|  | High | 58.3% |  | 79.2% |  |
| PDL1^+^ B cells | Low | 56.0% | 0.242 | 76.0% | 0.575 |
|  | High | 75.0% |  | 83.3% |  |
| PD1^+^ B cells | Low | 64.0% | 0.864 | 76.0% | 0.663 |
|  | High | 66.7% |  | 83.3% |  |
| TIM3^+^ memory B cells | Low | 60.0% | 0.630 | 72.0% | 0.257 |
|  | High | 70.8% |  | 87.5% |  |
| CD11c^+^ memory B cells | Low | 44.0% | **0.003** | 68.0% | **0.041** |
|  | High | 87.5% |  | 91.7% |  |
| LAG3^+^ memory B cells | Low | 64.0% | 0.774 | 84.0% | 0.563 |
|  | High | 66.7% |  | 75.0% |  |
| HLA-DR^+^ memory B cells | Low | 84.0% | **0.004** | 92.0% | **0.020** |
|  | High | 45.8% |  | 66.7% |  |
| CD24^+^ memory B cells | Low | 84.0% | **0.005** | 92.0% | **0.029** |
|  | High | 45.8% |  | 66.7% |  |
| CXCR5^+^ memory B cells | Low | 48.0% | **0.014** | 68.0% | 0.055 |
|  | High | 83.3% |  | 91.7% |  |
| CD73^+^ memory B cells | Low | 64.0% | 0.740 | 80.0% | 0.982 |
|  | High | 66.7% |  | 79.2% |  |
| PDL2^+^ memory B cells | Low | 60.0% | 0.537 | 68.0% | 0.058 |
|  | High | 70.8% |  | 91.7% |  |
| PDL1^+^ memory B cells | Low | 48.0% | **0.017** | 64.0% | **0.010** |
|  | High | 83.3% |  | 95.8% |  |
| PD1^+^ memory B cells | Low | 60.0% | 0.488 | 72.0% | 0.255 |
|  | High | 70.8% |  | 87.5% |  |
| TIM3^+^ naive B cells | Low | 72.0% | 0.272 | 80.0% | 0.845 |
|  | High | 58.3% |  | 79.2% |  |
| CD11c^+^ naive B cells | Low | 56.0% | 0.237 | 76.0% | 0.487 |
|  | High | 75.0% |  | 83.3% |  |
| LAG3^+^ naive B cells | Low | 64.0% | 0.751 | 76.0% | 0.612 |
|  | High | 66.7% |  | 83.3% |  |
| HLA-DR^+^ naive B cells | Low | 92.0% | **<0.001** | 96.0% | **0.002** |
|  | High | 37.5% |  | 62.5% |  |
| CD24^+^ naive B cells | Low | 72.0% | 0.400 | 80.0% | 0.880 |
|  | High | 58.3% |  | 79.2% |  |
| CXCR5^+^ naive B cells | Low | 48.0% | **0.018** | 68.0% | **0.041** |
|  | High | 83.3% |  | 91.7% |  |
| CD73^+^ naive B cells | Low | 80.0% | **0.036** | 88.0% | 0.103 |
|  | High | 50.0% |  | 70.8% |  |
| PDL2^+^ naive B cells | Low | 68.0% | 0.581 | 76.0% | 0.602 |
|  | High | 62.5% |  | 83.3% |  |
| PDL1^+^ naive B cells | Low | 52.0% | 0.075 | 72.0% | 0.202 |
|  | High | 79.2% |  | 87.5% |  |
| PD1^+^ naive B cells | Low | 64.0% | 0.847 | 76.0% | 0.622 |
|  | High | 66.7% |  | 83.3% |  |

**Supplementary Table 4.** Binary logistic regression analysis for 3 years DFS and OS in relation to expression of TIM3, CD11c, LAG3, HLA-DR, CD24, CXCR5, CD73, PDL1, PDL2, and PD-1 on B cells, memory B cells, and naive B cells in TDLNs. Significant p-values (p<0.05) are highlighted with bold numbers.

|  | DFS | | | OS | | |
| --- | --- | --- | --- | --- | --- | --- |
| Parameter | Variable | Univariate analysis odds ratio (95% CI) | P value | Variable | Univariate analysis odds ratio (95% CI) | P value |
| T-stage | T1-T2 | Ref. | **0.002** | Low | Ref. | **0.003** |
|  | T3-T4 | 7.944 (2.094-30.135) |  | High | 13.333 (2.389-74.404) |  |
| N-stage | N0 | Ref. | 0.301 | Low | Ref. | **0.016** |
|  | N+ | 1.875 (0.570-6.171) |  | High | 8.000 (1.481-43.202) |  |
| Smoking | Never | Ref. | **0.034** | Low | Ref. | **0.033** |
|  | Previous/Current | 4.179 (1.116-15.650) |  | High | 10.500 (1.211-91.026) |  |
| Proportion of Memory B cells | Low | Ref. | 0.241 | Low | Ref. | 0.439 |
|  | High | 2.118 (0.605-7.415) |  | High | 1.803 (0.405-8.026) |  |
| Proportion of naive B cells | Low | Ref. | 0.739 | Low | Ref. | 0.781 |
|  | High | 1.250 (0.336-4.655) |  | High | 1.243 (0.269-5.749) |  |
| Proportion of plasma B cells | Low | Ref. | 0.051 | Low | Ref. | **0.020** |
|  | High | 0.285 (0.081-1.004) |  | High | 0.077 (0.009-0.679) |  |
| TIM3^+^ B cells | Low | Ref. | 0.317 | Low | Ref. | 0.942 |
|  | High | 1.837 (0.558-6.049) |  | High | 1.053 (0.262-4.224) |  |
| CD11c^+^ B cells | Low | Ref. | **0.013** | Low | Ref. | 0.054 |
|  | High | 0.185 (0.049-0.698) |  | High | 0.193 (0.036-1.030) |  |
| LAG3^+^ B cells | Low | Ref. | 0.427 | Low | Ref. | 0.942 |
|  | High | 0.618 (0.188-2.029) |  | High | 1.053 (0.262-4.224) |  |
| HLA-DR^+^ B cells | Low | Ref. | **<0.001** | Low | Ref. | **0.016** |
|  | High | 19.167 (3.628-101.262) |  | High | 14.400 (1.653-125.410) |  |
| CD24^+^ B cells | Low | Ref. | 0.113 | Low | Ref. | 0.438 |
|  | High | 2.679 (0.791-9.071) |  | High | 1.750 (0.426-7.190) |  |
| CXCR5^+^ B cells | Low | Ref. | **0.013** | Low | Ref. | 0.054 |
|  | High | 0.185 (0.049-0.698) |  | High | 0.193 (0.036-1.030) |  |
| CD73^+^ B cells | Low | Ref. | 0.317 | Low | Ref. | 0.438 |
|  | High | 1.837 (0.558-6.049) |  | High | 1.750 (0.426-7.190) |  |
| PDL2^+^ B cells | Low | Ref. | 0.317 | Low | Ref. | 0.942 |
|  | High | 1.837 (0.558-6.049) |  | High | 1.053 (0.262-4.224) |  |
| PDL1^+^ B cells | Low | Ref. | 0.167 | Low | Ref. | 0.526 |
|  | High | 0.424 (0.126-1.430) |  | High | 0.633 (0.154-2.600) |  |
| PD1^+^ B cells | Low | Ref. | 0.845 | Low | Ref. | 0.526 |
|  | High | 0.889 (0.274-2.886) |  | High | 0.633 (0.154-2.600) |  |
| TIM3^+^ memory B cells | Low | Ref. | 0.427 | Low | Ref. | 0.188 |
|  | High | 0.618 (0.188-2.029) |  | High | 0.367 (0.083-1.633) |  |
| CD11c^+^ memory B cells | Low | Ref. | **0.003** | Low | Ref. | 0.054 |
|  | High | 0.112 (0.026-0.476) |  | High | 0.193 (0.036-1.030) |  |
| LAG3^+^ memory B cells | Low | Ref. | 0.845 | Low | Ref. | 0.438 |
|  | High | 0.889 (0.274-2.886) |  | High | 1.750 (0.426-7.190) |  |
| HLA-DR^+^ memory B cells | Low | Ref. | **0.007** | Low | Ref. | **0.041** |
|  | High | 6.205 (1.629-23.628) |  | High | 5.750 (1.076-30.720) |  |
| CD24^+^ memory B cells | Low | Ref. | **0.007** | Low | Ref. | **0.041** |
|  | High | 6.205 (1.629-23.628) |  | High | 5.750 (1.076-30.720) |  |
| CXCR5^+^ memory B cells | Low | Ref. | **0.013** | Low | Ref. | 0.054 |
|  | High | 0.185 (0.049-0.698) |  | High | 0.193 (0.036-1.030) |  |
| CD73^+^ memory B cells | Low | Ref. | 0.845 | Low | Ref. | 0.942 |
|  | High | 0.889 (0.274-2.886) |  | High | 1.053 (0.262-4.224) |  |
| PDL2^+^ memory B cells | Low | Ref. | 0.427 | Low | Ref. | 0.054 |
|  | High | 0.618 (0.188-2.029) |  | High | 0.193 (0.036-1.030) |  |
| PDL1^+^ memory B cells | Low | Ref. | **0.013** | Low | Ref. | **0.020** |
|  | High | 0.185 (0.049-0.698) |  | High | 0.077 (0.009-0.679) |  |
| PD1^+^ memory B cells | Low | Ref. | 0.427 | Low | Ref. | 0.188 |
|  | High | 0.618 (0.188-2.029) |  | High | 0.367 (0.083-1.633) |  |
| TIM3^+^ naive B cells | Low | Ref. | 0.317 | Low | Ref. | 0.942 |
|  | High | 1.837 (0.558-6.049) |  | High | 1.053 (0.262-4.224) |  |
| CD11c^+^ naive B cells | Low | Ref. | 0.167 | Low | Ref. | 0.526 |
|  | High | 0.424 (0.126-1.430) |  | High | 0.633 (0.154-2.600) |  |
| LAG3^+^ naive B cells | Low | Ref. | 0.845 | Low | Ref. | 0.526 |
|  | High | 0.889 (0.274-2.886) |  | High | 0.633 (0.154-2.600) |  |
| HLA-DR^+^ naive B cells | Low | Ref. | **<0.001** | Low | Ref. | **0.016** |
|  | High | 19.167 (3.628-101.262) |  | High | 14.400 (1.653-125.410) |  |
| CD24^+^ naive B cells | Low | Ref. | 0.317 | Low | Ref. | 0.942 |
|  | High | 1.837 (0.558-6.049) |  | High | 1.053 (0.262-4.224) |  |
| CXCR5^+^ naive B cells | Low | Ref. | **0.013** | Low | Ref. | 0.054 |
|  | High | 0.185 (0.049-0.698) |  | High | 0.193 (0.036-1.030) |  |
| CD73^+^ naive B cells | Low | Ref. | **0.032** | Low | Ref. | 0.147 |
|  | High | 4.000 (1.129-14.175) |  | High | 3.020 (0.678-13.442) |  |
| PDL2^+^ naive B cells | Low | Ref. | 0.686 | Low | Ref. | 0.526 |
|  | High | 1.275 (0.392-4.143) |  | High | 0.633 (0.154-2.600) |  |
| PDL1^+^ naive B cells | Low | Ref. | 0.051 | Low | Ref. | 0.188 |
|  | High | 0.285 (0.081-1.004) |  | High | 0.367 (0.083-1.633) |  |
| PD1^+^ naive B cells | Low | Ref. | 0.845 | Low | Ref. | 0.526 |
|  | High | 0.889 (0.274-2.886) |  | High | 0.633 (0.154-2.600) |  |
| Postoperative radiotherapy | No | Ref. | 0.160 | No | Ref. | N/A |
|  | Yes | 2.800 (0.665-11.791) |  | Yes | N/A* |  |
| SNB or Neck+SNLB | SNB | Ref. | 0.278 | SNB | Ref. | 0.810 |
|  | Neck+SNLB | 0.511 (0.152-1.716) |  | Neck+SNLB | 0.840 (0.202-3.490) |  |

* Binary logistic regression including postoperative radiotherapy was not calculated due to complete separation, as no deaths occurred in the group without postoperative radiotherapy.

| **Gate** | **Mean** | **SD** |
| --- | --- | --- |
| CD19+CD20+ of Total Cells | 18 % | 7.59 |
| HLA-DR+ B cells | 96 % | 2.23 |
| Live cells, B cells | 89 % | 11.5 |

**Supplementary figure 1.** Gating strategy for B cells, memory B cells, naïve B cells, and plasma cells. The mean proportions and SD of the first four gates are presented in the table. Each proportion is the proportion of the parent gate above, for example, 89% of the HLA-DR+ cells were live cells.

**Supplementary Figure 2.** An exemplary gating of positive populations on B cells for the investigated markers.

**Supplementary figure 3. Comparison of surface expression of CD11c (A), CD24 (B), CXCR5 (C), CD73 (D), HLA-DR (E), PD-1 (F), PDL1 (G), PDL2 (H), LAG3 (I) and TIM3 (J) on memory (blue) and naïve (red) B cells from TDLNs in patients with recurrent and non-recurrent disease.** A two-way ANOVA was performed. Significance is shown by asterisk symbols with significance levels as follows: *p<0.05, **p<0.001, ***p<0.0001.

**Supplementary figure 4. Comparison of surface expression of CD11c (A), CD24 (B), CXCR5 (C), CD73 (D), HLA-DR (E), PD-1 (F), PDL1 (G), PDL2 (H), Tim3 (I) and proportion of B cell phenotypes (J) on recurrent patients (n=17) without metastasis, N0 (blue) and with metastasis, N1 (red) B cells from TDLNs.** Mann-Whitney test with correction for multiple comparisons using the Holm-Šídák test was performed. Not significant is shown by ns.

**Supplementary Methods**

*Cryo-preserved cells*

1. Use DMEM + 10% FBS and DMEM + 10%FBS + 20% DMSO
2. Dilute cells in 500µl DMEM + 10% FBS per cryo tube, add dropwise DMEM + 10%FBS + 20% DMSO, and double the volume.
3. Transfer 1 ml cell suspension onto a cryo tube.
4. Freeze cells in Mr. Frosty (-1°C/min) at -80 °C and for long-term storage at -180 °C.

*Thaw cells*

1. Warm the cryo tube in a water bath at 40oc until it almost thawed.
2. Transfer cell suspension into a 15ml falcon tube with 10ml warm media (DMEM + 5% FBS).
3. Wash cells two times with PBS, 300g, 5min.

*Flow cytometry Instrument setup and compensation*Purified lymphocytes were used to adjust the instrument voltage to 2.5 x rSDen. FACS Diva application settings were then used to reduce PMT variability over time; for the daily tracking of the LSR Fortessa, cytometer set-up and tracking beads (BD Biosciences, New Jersey, USA) were used daily before sample acquisition. Compensation was performed in FACSdiva Software with single stained anti-mouse beads (BD Biosciences, New Jersey, USA), anti-rat beads (BD Biosciences, New Jersey, USA), and ArC reactive beads (Thermo Scientific, Massachusetts, USA, #A10346).

*Quality control*

The mean frequency of live B cells were 76 % with a standard deviation of 15 which was deemed acceptable.
